# Ferroptosis involves in Schwann cell death in diabetic peripheral neuropathy

**DOI:** 10.1515/med-2023-0809

**Published:** 2023-10-09

**Authors:** Kai-yan Wu, Fei Deng, Xin-yu Mao, Dan Zhou, Wei-gan Shen

**Affiliations:** Department of Cell Biology, School of Medicine of Yangzhou University, Yangzhou, Jiangsu, 225009, China; Department of Central Laboratory, Jintan Hospital, Jiangsu University, 500 Avenue Jintan, Jintan, Jiangsu, 213200, China

**Keywords:** ferroptosis, diabetic peripheral neuropathy, Schwann cell, NRF2 signaling pathway

## Abstract

Accumulating evidence shows that Schwann cells’ (SCs) death caused by high glucose (HG) is involved in the pathological process of diabetic peripheral neuropathy (DPN). Ferroptosis is a novel form of regulatory cell death driven by iron-dependent lipid peroxidation. However, it is not clear whether ferroptosis is involved in the death process of SCs induced by HG. The expression of ferroptosis-related indicators in the serum of DPN patients was detected by ELISA. Subsequently, using cell counting kit‑8, western blot, real-time PCR, and Ki-67 staining, we investigated the effects of HG on the ferroptosis of SCs and initially explored the underlying mechanism. The results showed that the serum levels of glutathione peroxidase 4 (GPX4) and glutathione in patients with DPN decreased, while malondialdehyde levels increased significantly. Then, we observed that erastin and HG induced ferroptosis in SCs, resulting in the decrease in cell activity and the expression level of GPX4 and SLC7A11, which could be effectively reversed by the ferroptosis inhibitor Fer-1. Mechanistically, HG induced ferroptosis in SCs by inhibiting the NRF2 signaling pathway. Our results showed that ferroptosis was involved in the death process of SCs induced by HG. Inhibition of ferroptosis in SCs might create a new avenue for the treatment of DPN.

## 1 Introduction

Diabetic peripheral neuropathy (DPN), one of the most common chronic complications of diabetes, affects up to 50% of diabetic patients [1]. Nowadays, clinical treatment for DPN mainly focuses on strict hypoglycemic therapy and pain management, while a restricted number of patients benefit from it, which means more culprits besides hyperglycemia may participate in the process of DPN [2]. Accumulating evidence indicates that the death of Schwann cells (SCs) induced by hyperglycemia is involved in the pathogenesis of DPN, including oxidative stress, inflammatory response, autophagy dysfunction, ERS, and other pathological processes [3]. In particular, during DPN, the death of SCs will lead to myelin destruction, demyelination, axonal conduction abnormalities, and impaired neuronal regeneration, thus accelerating the progress of DPN [4]. Therefore, understanding the death mechanism of SCs under hyperglycemia may be an important entry point for the treatment of DPN.

Programmed cell death is strictly regulated by complex intracellular and extracellular signals, which is very important for diverse various biological processes, including mammalian development, homeostasis, and disease [5]. Ferroptosis is a novel form of programmed cell death, which is different from the traditional cognitive cell death modes [6]. The key mediators of ferroptosis are glutathione peroxidase 4 (GPX4) and SLC7A11, a functional subunit of cystine/glutamate reverse transport system Xc^−^. Low levels of SLC7A11 and GPX4 inactivation reduce glutathione (GSH) synthesis and lipid peroxide degradation, respectively. Both of these changes cause the accumulation of lipid peroxides, which in turn leads to the occurrence of cell ferroptosis [7,8,9]. Recent studies revealed that HG can induce ferroptosis in osteoblasts via increased intracellular ROS and the accumulation of lipid oxides and GSH depletion in diabetic osteoporosis [10]. During DPN, the enhancement of oxidative stress and reactive oxygen species (ROS) are dominant features that lead to SCs’ death, while ferroptosis is a cell death mode closely related to ROS [11,12,13]. Therefore, we speculate that ferroptosis may be involved in the death of SCs induced by HG.

In this study, the aim was to explore the role of ferroptosis in HG-induced SCs’ death. To test our hypothesis, we used HG-stimulated cultured SCs in vitro. Furthermore, we sought to determine the possible associated mechanism.

## 2 Materials and methods

### 2.1 Baseline data

From January 2021 to August 2021, DPN patients (*n* = 65) who underwent treatment and healthy volunteers (*n* = 23) who underwent physical examination in the Jintan Hospital affiliated to Jiangsu University were recruited for our research. Healthy volunteers were selected as a control group. Clinical data were recorded for all participants including gender, age, and diabetes mellitus (DM) duration. Inclusion criteria for DPN were as follows: (1) met the diagnostic criteria for DM and (2) the symptoms of DPN met the diagnostic criteria in the Chinese Guidelines for the Prevention and Treatment of Type 2 DM (2020 edition) [14]. Exclusion criteria were as follows: (1) patients with other forms of neuropathy, including neuropathy caused by drugs, cervical and lumbar spine lesions, Guillain–Barré syndrome, and other causes; (2) those with gestational diabetes; (3) those with tumor; (4) those with serious diseases of other organ systems in combination; and (5) those with mental disorders, hearing impairment, and cognitive dysfunction. This study was approved by the Ethics Review Committee of Jintan Hospital affiliated to Jiangsu University (no. LS2021009). All participants were informed of the study details and provided informed consent.

### 2.2 Detection of biochemical parameters

Whole blood was collected from the elbow vein of the participants who fasted overnight using a vacuum blood collection tube with anticoagulant or without anticoagulant. The whole blood containing anticoagulant was used to detect hemoglobin A1c (HbA1c) using a Lifotronic H9 (Lifotronic Technology Co., Ltd, Shenzhen, China). When whole blood without anticoagulants coagulated, the serum was separated by centrifugation at 3,000 rpm for 5 min. Serum levels of glucose were estimated using a LABOSPECT 008AS Automatic Chemical Analyzer (Hitachi, Tokyo, Japan). Human ELISA kit (Bios-wamp, Wuhan, China) was used to quantify serum levels of GPX4, GSH, and malondialdehyde (MDA) in patients with DPN and volunteers. All analysis steps were performed according to the manufacturer’s instructions.

### 2.3 Vibration perception threshold measurement

Vibration perception threshold (VPT) was assessed using a Sensiometer A100 (Laxons Technology Co., Ltd, Beijing, China). The patient was informed and familiar with the vibration sensation, and then, the professional performed the operation according to the manufacturer’s instructions. Each foot of the patient was repeated for 3 times, and the average VPT of both feet was recorded. In addition, according to the test value, the risk of diabetic foot is predicted. 0–10, 10–15, 15–25, and more than 25 V are considered normal, light risk, middle risk and severe risk, respectively.

### 2.4 Cell culture and treatment

SC line RSC96 was purchased from the cell bank of the China Academy of Sciences (Shanghai, China). The cell line was cultured in DMEM (Gibco, Grand Island, USA) supplemented with 10% fetal bovine serum (Gibco) and 1% pen/strep (Gibco), and incubated at 5% CO_2_ and 37°C.

To establish an HG cell model RSC96 was treated with DMEM containing 100 mM glucose for 48 h as previously described [15]. Cells were cultured to logarithmic phase and grouped as follows: normal control group (DMEM containing 25 mM glucose), high-glucose group (DMEM containing 100 mM glucose), Erastin (Selleckchem, Houston, USA) group (DMEM containing 25 mM glucose and 2 μM Erastin), and the ferroptosis inhibitor ferrostatin-1 (Fer-1) (Selleckchem) group (DMEM containing 100 mM glucose and 10 μM Fer-1). Erastin, as an inducer of cell ferroptosis, was used as a positive control.

### 2.5 Cell viability assay

Cell viability was analyzed using the cell counting kit-8 (CCK-8) (Biosharp, Beijing, China). RSC96 cells under different treatment conditions were seeded in 96-well plates (5  ×  10^3^ cells per well) and cultured for 48 h. Then, 10 μL of CCK-8 solution was added to RSC96 cells for incubation for 1.5 h. The absorbance of the cells in each well at 450 nm was measured on a microplate reader (Multiskan FC, Thermoscientific, Waltham, USA).

### 2.6 Cell transfection

NRF2 gene expression was knocked down by transfecting RSC96 cells with siRNA-NRF2 or siRNA control (GenePharma Co., Ltd., Shanghai, China). Transient siRNA transfection was performed using Lipofectamine 2000 (Invitrogen, Carlsbad, CA, USA), according to the manufacturer’s instructions. After 24 h of transfection, SCs were treated with HG and Fer-1. The transfection efficiency was verified by real-time PCR and western blotting.

### 2.7 Immunofluorescence staining

Briefly, RSC96 cells under different treatment conditions were fixed with 4% paraformaldehyde at 4°C for 30 min. Subsequently, paraformaldehyde was removed and cells were permeabilized with 0.5% Triton X-100 and blocked with 5% BSA in PBS. Then, the cells were incubated with Ki-67 rabbit monoclonal antibody (1:200 dilution, Servicebio, Wuhan, China) at 4°C overnight, followed by washing and incubation with Cy3-conjugated secondary antibody (1:200 dilution, Servicebio) at 37 °C for 1 h. The cells were stained with DAPI nuclear stain (5 μg/mL) for 5–10 min. The images were taken using a CK-X53 fluorescent microscope (Olympus, Tokyo, Japan).

### 2.8 Western blotting

Western blot analysis was performed as previously described [16]. Briefly, the collected cells were lysed with RIPA to extract protein. Afterwards, the samples were transferred to nitrocellulose filter membranes and 5% skim milk powder solution, and then, the primary antibody (GPX4, SLC7A11, NQO1, HO-1, 1:1,000 dilution, Abcam, Cambridge, USA; NRF2, 1:1,000 dilution, Beyotime, China) was incubated at 4°C overnight. The membranes were then washed and incubated with the secondary antibody. β-Actin (1:2,000 dilution, CST, Danvers, USA) was used as an internal control.

### 2.9 RNA isolation and quantitative real-time PCR

Total RNA was extracted from cells. cDNA was synthesized using All-in-One™ First-Strand cDNA Synthesis Kit (Genecopoeia, Germantown, USA) according to the manufacturer’s protocols. qRT-PCR analysis was performed with All-in-One™ qPCR Mix (Genecopoeia). The primers were purchased from Genecopoeia. Real-time PCR was performed using a Quant Gene 9600 system (Bioer, Hangzhou, China). The relative expression of mRNA was evaluated by the 2^−ΔΔCt^ method and normalized to GAPDH.

### 2.10 Statistical analysis

Statistical analyses were performed using the GraphPad Prism software (Version 5.0, GraphPad, San Diego, CA, USA). Data were presented as mean ± standard error of the mean. The groups were compared using the Student’s *t* test and one-way analysis of variance. Results were considered statistically significant for *P* values less than 0.05.

## 3 Results

### 3.1 Characteristics of baseline data

The baseline characteristics of the DPN group and the control group are shown in Table 1. The age of the DPN and control groups was 62.25 ± 11.48 (median age 59 (34–85)) and 26.30 ± 13.90 (median age 21 (15–75) years, respectively. The mean DM duration of the DPN group was 9.60 ± 6.49 years. When comparing the control group, GLU and HbA1c in the DPN group tended to have higher levels.

**Table 1:** Clinical characteristics

Characteristics	Control group (*n* = 23)	DPN group (*n* = 65)
Gender, *n* (%)		
Male	17 (73.91)	40 (61.54)
Female	6 (26.09)	25 (38.46)
Age (years)	26.30 ± 13.90	62.25 ± 11.48
GLU (mmol/L)	4.95 ± 0.34	9.37 ± 3.39
HbA1c (%)	4.90 ± 0.20	9.47 ± 2.16

GLU, glucose; HbA1C, hemoglobin A1c.

### 3.2 The level of ferroptosis elevated in DPN patients

**Figure 1:**
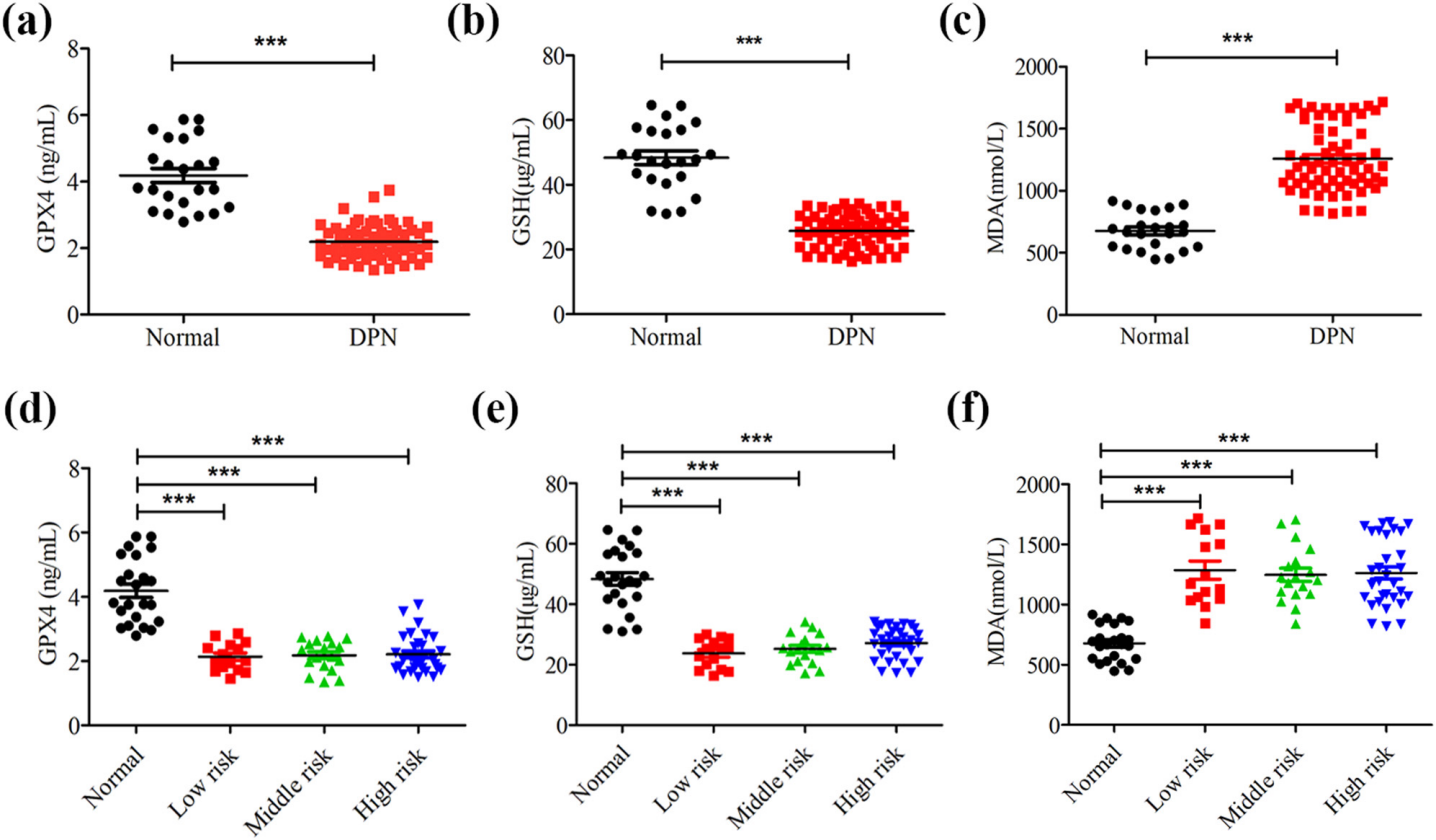
The level of ferroptosis elevated in DPN patients. (a–c) ELISA was used to detect the serum level of ferroptosis-related index, including GPX4, GSH, and MDA. (d–f) Expression level of GPX4, GSH, and MDA in different risk stratification. ***P < 0.001.

We first measured the indicators directly related to ferroptosis in the serum of DPN patients and healthy volunteers. The results showed that GPX4 and GSH were decreased significantly, and lipid peroxide MDA was markedly increased in the serum of patients with DPN compared with those in the serum of healthy volunteers, (Figure 1a–c). Given that the detection results of VPT can not only be used to diagnose DPN, but also predict the risk of diabetic foot, we further evaluated the differences in these related indicators of ferroptosis under different risk stratification. The data showed that GSH, GPX4 and MDA in different risk stratification were statistically significant compared with the control group, but there was no statistical difference between risk stratification groups (Figure 1d–f). These results suggest that the level of ferroptosis is increased in the serum of DPN patients, which is not directly related to the degree of risk of diabetic foot predicted by VPT.

### 3.3 HG induced the ferroptosis of SCs

**Figure 2:**
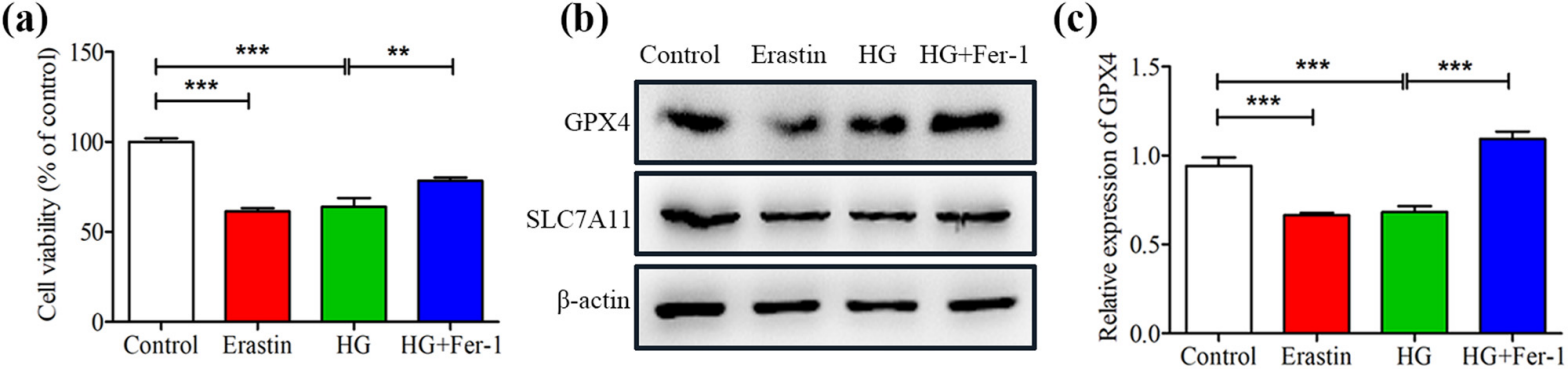
High glucose-induced ferroptosis in SCs. SCs were cultured and treated with eratin and fer-1 combined with high glucose. (a) CCK-8 assay was performed to estimate the cell proliferation viability of SCs. (b) The Ferroptosis-related protein levels of GPX4 and SLC7A11 were determined by western blotting. (c) Real-time PCR analysis of the expression of GPX4. **P < 0.001, ***P < 0.0001.

We constructed an in vitro model of SCs induced by HG. Consistent with previously reported [15], HG effectively inhibited the proliferation activity of SCs (Figure 2a). To determine whether the decrease of the proliferation activity of SCs induced by HG was due to ferroptosis. Erastin, a classic activator of ferroptosis, was used to treat SCs. We found that the trend of erastin inhibiting SCs’ proliferation activity was similar to that induced by HG, while Fer-1 (a specific inhibitor of ferroptosis) reversed the effect of HG-induced decrease in the proliferation activity of SCs (Figure 2a). Importantly, treatment of HG-induced SCs with Fer-1 significantly increased the expression of GPX4 and SLC7A11, which resisted the key regulatory components of cell ferroptosis (Figure 2b and Figure S1). Moreover, qRT-PCR analysis showed that the level of GPX4 in SCs exposed to erastin and glucose decreased; whereas Fer-1 increased the level of GPX4 in HG-treated SCs (Figure 2c). Taken together, these results suggest that HG could induce ferroptosis in SCs.

### 3.4 HG-induced ferroptosis in SCs by inhibiting NRF2 signaling pathway

**Figure 3:**
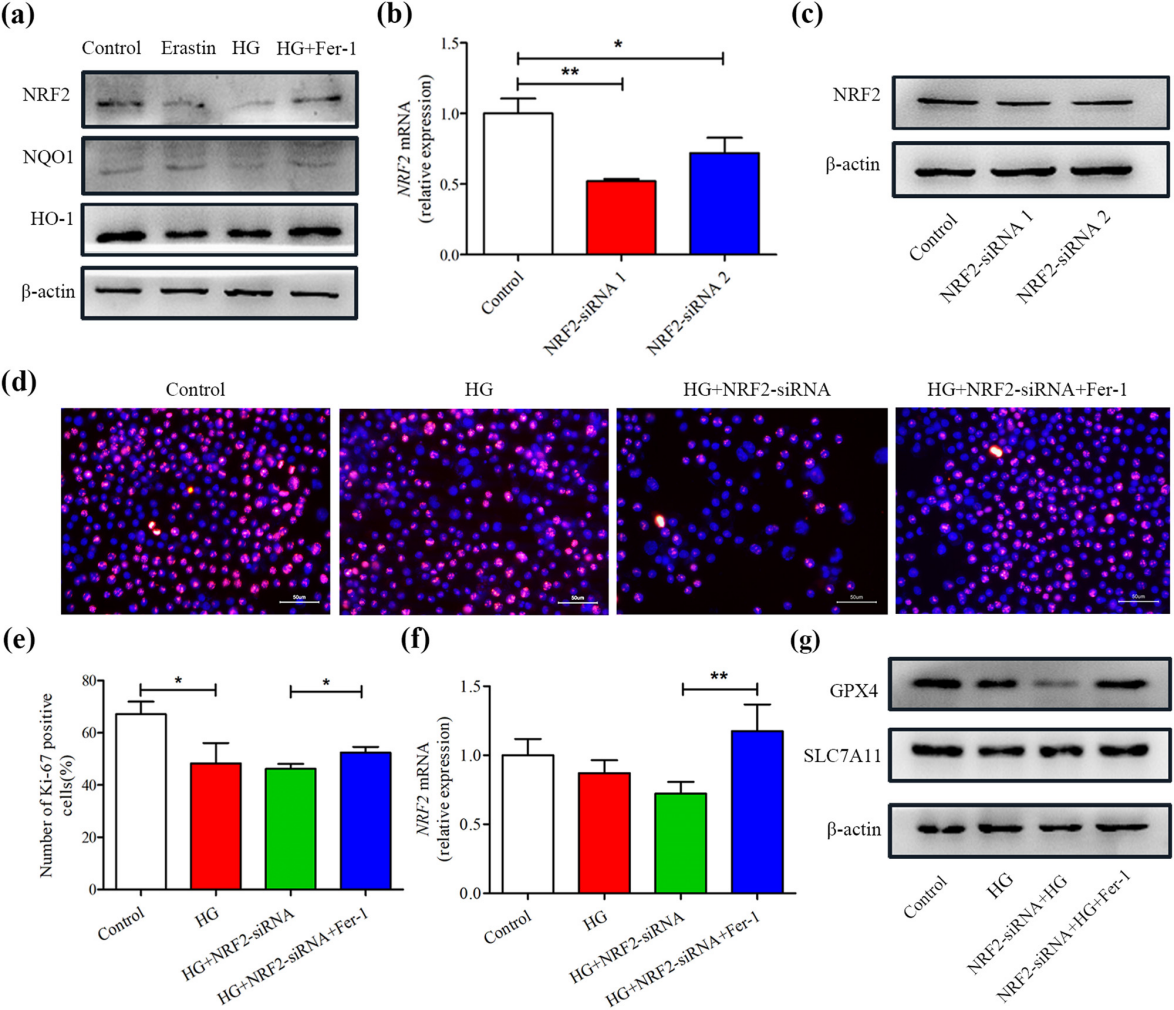
High glucose-induced ferroptosis via inhibiting NRF2 signaling pathway. (a) The protein levels of NRF2, NQO1, and HO-1 were determined using western blot assays. (b) Expression of NRF2 level in SCs with NRF2 knock-down was assessed by real-time PCR and Western blot (c). (d and e) Representative images and quantification data of Ki67 staining. Scale bar: 50 μm. (f) Real-time PCR analysis of NRF2 expression in SCs after treatments. (g) GPX4 and SLC7A11 protein levels were determined by western blot in each group. *P < 0.05, **P < 0.001.

The typical characteristic of ferroptosis is the overproduction of lipid ROS. However, Previous research has established that HG can induce SCs’ oxidative stress, that is, ROS production increase and antioxidant protein decrease [17]. NRF2 signaling pathway is the direct downstream pathway of ROS, which regulates the transcription of ARE-dependent genes to balance the oxidation medium and maintain the redox homeostasis of cells [18]. The levels of NRF2, NQO1, and HO-1 were inhibited during HG or erastin-induced ferroptosis, as shown by western blotting. However, these proteins’ expression was significantly increased by treatment with Fer-1 (Figure 3a and Figure S2), suggesting that the NRF2 signaling pathway is involved in the process of ferroptosis in SCs induced by HG. To further determine the role of the NRF2 pathway in HG-induced ferroptosis, SCs were transiently transfected with NRF2-siRNA or NC-siRNA, which led to a decrease in NRF2 expression in the SCs Figure 3b and c and Figure S3. Due to NRF2-siRNA1 having the best knock-down efficiency, we chose it for SCs’ transfection and then stimulated with HG. As shown in Figure 3d and e, HG significantly inhibited the proliferation of SCs transfected with NRF2-siRNA, but this detrimental effect could be strongly reversed by Fer-1. The beneficial effect of Fer-1 may be achieved by improving the expression of NRF2 (Figure 3f). Additionally, HG-treated SCs with NRF2-siRNA transfected aggravated the down-regulation of GPX4 and SLCA711 compared with the HG group, whereas this was alleviated in the Fer-1 treatment group Figure 3g and Figure S4, suggesting that the mechanism of HG-induced ferroptosis in SCs may be through inhibiting NRF2 signal pathway.

## 4 Discussion

DPN, as the most common complication of diabetes, is characterized by the impaired nerve fibers undergoing segmental demyelination [1,19]. As supporting cells in the peripheral nervous system, the death of SCs under hyperglycemia has been considered the underlying cause of demyelination [20,21]. Therefore, understanding the death of SCs may help to reveal their mechanism in the associated loss of the myelin sheath in DPN. However, most previous studies on SCs’ death under diabetic conditions mainly focused on apoptotic cell death [17,22,23]. In recent years, some emerging cell death modes, including necroptosis [24], pyroptosis [25], and autophagy [26], have made contributions to the cognition of SCs death. In this study, we further demonstrated that ferroptosis involves in SCs’ death in DPN.

Previous studies have shown that demyelination is a significant feature of DPN pathological changes [27]. In the peripheral nervous system, axon demyelination is closely related to the abnormality of SCs, because the formation of myelin sheath depends on the development and maturity of SC to wrap the axon [28]. In addition, SCs can provide abundant neurotrophic factors and oxygen support for axons [28]. Under the condition of hyperglycemia, the production of ROS is higher than that of the formation of antioxidants, which will lead to the imbalance of redox homeostasis. The imbalance of redox homeostasis elicits the peroxidation of protein, lipid, and nucleic acid and further triggers the over-activation of oxidative stress, resulting in metabolic dysfunction and death of SCs [17]. Consistent with this, our results showed that antioxidants GPX4 and GSH were significantly reduced in the serum of DPN patients, while MDA, an indicator closely related to lipid peroxidation, was significantly increased. Lipid peroxidation is considered to induce ferroptosis, a new type of programmed cell death, which is characterized by the increase in iron content in cells and the increase in ROS production caused by GSH depletion, leading to lipid peroxidation and eventually cell death [7,29,30]. This implies that the increase in ROS concentration in SCs may induce ferroptosis, leading to myelin damage, even demyelination. In this study, we observed that the decrease in SCs’ viability induced by HG exhibited the similar effects to erastin, while Fer-1 could reverse this harmful effect. Moreover, Fer-1 also increased the expression of GPX4 and SLC7A11 in SCs induced by HG. These results confirmed the occurrence of ferroptosis in SCs induced by HG which may help us understand the mechanism of axonal demyelination in DPN. A recent study is similar to our results, that is, human herpesvirus 7 mediates the increase of ROS production by up-regulating Cox4i2, which results in SCs ferroptosis and the myelin sheath damage of facial nerve, while inhibiting the ferroptosis can promote the maturity of SCs and allow the nerve to form a more morphological myelin sheath [31,32]. Therefore, we speculate that reversing the ferroptosis of SCs may contribute to myelin regeneration during DPN.NRF2, a well-known transcription factor, has been identified as the main regulator of the defensive response to oxidative stress [33]. During oxidative stress, NRF2 dissociates from the heterodimer of Keap1 in the cytosol, allowing NRF2 to translocate to the nucleus, where NRF2 interacts with ARE to induce the expression of the downstream antioxidant gene (including HO-1, NQO1, etc.) to maintain oxidative stress homeostasis in the cell [18,34]. In fact, HG can induce the excessive production of ROS in cells, which leads to peroxidation and the collapse of NRF2 antioxidant system [35,36]. It is worth noting that many protein enzymes responsible for preventing lipid peroxidation and causing ferroptosis are NRF2 target genes [18,37]. It is reminiscent that HG by inhibiting the NRF2 signaling pathway may initiate the cellular suicide procedure, especially ferroptosis. Therefore, we put forward a reasonable hypothesis that HG-induced ferroptosis in SCs may be achieved by inhibiting the NRF2 signaling pathway. As predicted, HG-treated SCs exhibited decreased NRF2 as well as its downstream targets including HO-1 and NQO1. Moreover, when NRF2 was knocked down, HG aggravated the inhibition effect of SCs proliferation and decreased the expression of GPX4 and SLC7A11 in SCs, whereas Fer-1 alleviated these outcomes. Overall, these findings reveal that HG induced ferroptosis in SCs by inhibiting NRF2 pathway.

There are still some limitations of this study which are as follows: (1) only the indicators directly related to ferroptosis in the serum of DPN patients and healthy volunteers were detected in this experiment, and the serum of diabetic patients needs to be detected and further evaluated. (2) *In vivo* experiments have not been carried out, and animal experiments need to be further verified to enrich our findings.

## 5 Conclusion

**Figure 4:**
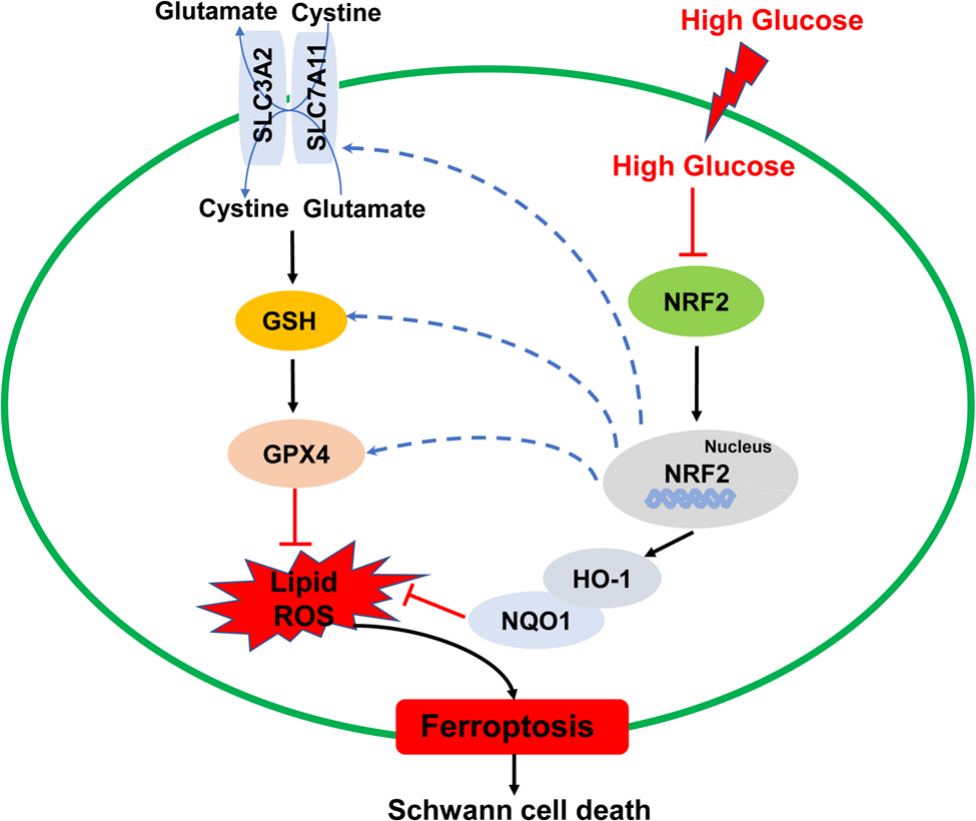
A schematic diagram of proposed molecular mechanisms of ferroptosis induced by high glucose in SCs. HG may induce ferroptosis in SCs by inhibiting NRF2 pathway and downstream ferroptosis-related proteins (SLC7A11, GSH, and GPX4).

In summary, the results of the present study provide evidence that HG induced ferroptosis in SCs by inhibiting the NRF2 pathway. From the perspective of ferroptosis, we describe a new mechanism of SCs death during DPN (Figure 4). More importantly and practically, targeting SCs ferroptosis may be a promising treatment strategy for DPN.

## Supplementary Material

Supplementary Figure

## References

[1] Barrell K, Smith AG. Peripheral neuropathy. Med Clin North Am. 2019;103(2):383–97. 10.1016/j.mcna.2018.10.006 30704689

[2] Stino AM, Rumora AE, Kim B, Feldman EL. Evolving concepts on the role of dyslipidemia, bioenergetics, and inflammation in the pathogenesis and treatment of diabetic peripheral neuropathy. J Peripher Nerv Syst. 2020;25(2):76–84. 10.1111/jns.12387 PMC7375363 32412144

[3] Li J, Guan R, Pan L. Mechanism of Schwann cells in diabetic peripheral neuropathy: A review. Medicine (Baltimore). 2023;102(1):e32653. 10.1097/MD.0000000000032653 PMC9829292 36607875

[4] Goncalves NP, Vaegter CB, Andersen H, Ostergaard L, Calcutt NA, Jensen TS. Schwann cell interactions with axons and microvessels in diabetic neuropathy. Nat Rev Neurol. 2017;13(3):135–47. 10.1038/nrneurol.2016.201 PMC7391875 28134254

[5] Moujalled D, Strasser A, Liddell JR. Molecular mechanisms of cell death in neurological diseases. Cell Death Differ. 2021;28(7):2029–44. 10.1038/s41418-021-00814-y PMC8257776 34099897

[6] Stockwell BR, Friedmann Angeli JP, Bayir H, Bush AI, Conrad M, Dixon SJ, et al. Ferroptosis: A regulated cell death nexus linking metabolism, redox biology, and disease. Cell. 2017;171(2):273–85. 10.1016/j.cell.2017.09.021 PMC5685180 28985560

[7] Xie Y, Hou W, Song X, Yu Y, Huang J, Sun X, et al. Ferroptosis: Process and function. Cell Death Differ. 2016;23(3):369–79. 10.1038/cdd.2015.158 PMC5072448 26794443

[8] Yang WS, Stockwell BR. Ferroptosis: Death by lipid peroxidation. Trends Cell Biol. 2016;26(3):165–76. 10.1016/j.tcb.2015.10.014 PMC4764384 26653790

[9] Ursini F, Maiorino M. Lipid peroxidation and ferroptosis: The role of GSH and GPx4. Free Radic Biol Med. 2020;152:175–85. 10.1016/j.freeradbiomed.2020.02.027 32165281

[10] Ma H, Wang X, Zhang W, Li H, Zhao W, Sun J, et al. Melatonin suppresses ferroptosis induced by high glucose via activation of the Nrf2/HO-1 signaling pathway in type 2 diabetic osteoporosis. Oxid Med Cell Longev. 2020;2020:9067610. 10.1155/2020/9067610 PMC7732386 33343809

[11] Pop-Busui R, Sima A, Stevens M. Diabetic neuropathy and oxidative stress. Diabetes Metab Res Rev. 2006;22(4):257–73. 10.1002/dmrr.625 16506271

[12] Vincent AM, Brownlee M, Russell JW. Oxidative stress and programmed cell death in diabetic neuropathy. Ann N Y Acad Sci. 2002;959:368–83. 10.1111/j.1749-6632.2002.tb02108.x 11976211

[13] Cao JY, Dixon SJ. Mechanisms of ferroptosis. Cell Mol Life Sci. 2016;73(11–12):2195–209. 10.1007/s00018-016-2194-1 PMC4887533 27048822

[14] Society C. Guideline for the prevention and treatment of type 2 diabetes mellitus in China (2020 edition). Chin J Diabetes Mellitus. 2021;13(4):315–409.

[15] Xu DD, Li WT, Jiang D, Wu HG, Ren MS, Chen MQ, et al. 3-N-Butylphthalide mitigates high glucose-induced injury to Schwann cells: Association with nitrosation and apoptosis. Neural Regen Res. 2019;14(3):513–8. 10.4103/1673-5374.245590 PMC6334601 30539821

[16] Ma Y, Dong L, Zhou D, Li L, Zhang W, Zhen Y, et al. Extracellular vesicles from human umbilical cord mesenchymal stem cells improve nerve regeneration after sciatic nerve transection in rats. J Cell Mol Med. 2019;23(4):2822–35. 10.1111/jcmm.14190 PMC6433678 30772948

[17] Li R, Wang B, Wu C, Li D, Wu Y, Ye L, et al. Acidic fibroblast growth factor attenuates type 2 diabetes-induced demyelination via suppressing oxidative stress damage. Cell Death Dis. 2021;12(1):107. 10.1038/s41419-021-03407-2 PMC7819983 33479232

[18] Dodson M, Castro-Portuguez R, Zhang DD. NRF2 plays a critical role in mitigating lipid peroxidation and ferroptosis. Redox Biol. 2019;23:101107. 10.1016/j.redox.2019.101107 PMC6859567 30692038

[19] Malik RA. Pathology of human diabetic neuropathy. Handb Clin Neurol. 2014;126:249–59. 10.1016/B978-0-444-53480-4.00016-3 25410227

[20] Mizisin AP, Shelton GD, Wagner S, Rusbridge C, Powell HC. Myelin splitting, Schwann cell injury and demyelination in feline diabetic neuropathy. Acta Neuropathol. 1998;95(2):171–4. 10.1007/s004010050783 9498053

[21] Naruse K. Schwann cells as crucial players in diabetic neuropathy. Adv Exp Med Biol. 2019;1190:345–56. 10.1007/978-981-32-9636-7_22 31760655

[22] Liu YP, Shao SJ, Guo HD. Schwann cells apoptosis is induced by high glucose in diabetic peripheral neuropathy. Life Sci. 2020;248:117459. 10.1016/j.lfs.2020.117459 32092332

[23] Zhang D, Chang S, Li X, Shi H, Jing B, Chen Z, et al. Therapeutic effect of paeoniflorin on chronic constriction injury of the sciatic nerve via the inhibition of Schwann cell apoptosis. Phytother Res. 2022;36(6):2572–82. 10.1002/ptr.7472 PMC9320937 35499270

[24] Belavgeni A, Maremonti F, Stadtmuller M, Bornstein SR, Linkermann A. Schwann cell necroptosis in diabetic neuropathy. Proc Natl Acad Sci U S A. 2022;119(17):e2204049119. 10.1073/pnas.2204049119 PMC9170031 35446622

[25] Cheng YC, Chu LW, Chen JY, Hsieh SL, Chang YC, Dai ZK, et al. Loganin attenuates high glucose-induced schwann cells pyroptosis by inhibiting ROS generation and NLRP3 inflammasome activation. Cells. 2020;9(9):1948. 10.3390/cells9091948 PMC7564733 32842536

[26] Choi SJ, Kim S, Lee WS, Kim DW, Kim CS, Oh SH. Autophagy dysfunction in a diabetic peripheral neuropathy model. Plast Reconstr Surg. 2023;151(2):355–64. 10.1097/PRS.0000000000009844 36355029

[27] Jaffey PB, Gelman BB. Increased vulnerability to demyelination in streptozotocin diabetic rats. J Comp Neurol. 1996;373(1):55–61. 10.1002/(SICI)1096-9861(19960909)373:1<55::AID-CNE5>3.0.CO;2-C 8876462

[28] Jessen KR, Mirsky R. The origin and development of glial cells in peripheral nerves. Nat Rev Neurosci. 2005;6(9):671–82. 10.1038/nrn1746 16136171

[29] Dixon SJ, Lemberg KM, Lamprecht MR, Skouta R, Zaitsev EM, Gleason CE, et al. Ferroptosis: An iron-dependent form of nonapoptotic cell death. Cell. 2012;149(5):1060–72. 10.1016/j.cell.2012.03.042 PMC3367386 22632970

[30] Ren JX, Sun X, Yan XL, Guo ZN, Yang Y. Ferroptosis in neurological diseases. Front Cell Neurosci. 2020;14:218. 10.3389/fncel.2020.00218 PMC7370841 32754017

[31] Chang B, Guan H, Wang X, Chen Z, Zhu W, Wei X, et al. Cox4i2 triggers an increase in reactive oxygen species, leading to ferroptosis and apoptosis in HHV7 infected Schwann cells. Front Mol Biosci. 2021;8:660072. 10.3389/fmolb.2021.660072 PMC8138133 34026834

[32] Gao D, Huang Y, Sun X, Yang J, Chen J, He J. Overexpression of c-Jun inhibits erastin-induced ferroptosis in Schwann cells and promotes repair of facial nerve function. J Cell Mol Med. 2022;26(8):2191–204. 10.1111/jcmm.17241 PMC8995448 35191156

[33] Tonelli C, Chio IIC, Tuveson DA. Transcriptional regulation by Nrf2. Antioxid Redox Signal. 2018;29(17):1727–45. 10.1089/ars.2017.7342 PMC6208165 28899199

[34] Pasupuleti VR, Arigela CS, Gan SH, Salam SKN, Krishnan KT, Rahman NA, et al. A review on oxidative stress, diabetic complications, and the roles of honey polyphenols. Oxid Med Cell Longev. 2020;2020:8878172. 10.1155/2020/8878172 PMC7704201 33299532

[35] Kumar A, Mittal R. Nrf2: A potential therapeutic target for diabetic neuropathy. Inflammopharmacology. 2017;25(4):393–402. 10.1007/s10787-017-0339-y 28353124

[36] Gupta A, Behl T, Sehgal A, Bhatia S, Jaglan D, Bungau S. Therapeutic potential of Nrf-2 pathway in the treatment of diabetic neuropathy and nephropathy. Mol Biol Rep. 2021;48(3):2761–74. 10.1007/s11033-021-06257-5 33754251

[37] Shakya A, McKee NW, Dodson M, Chapman E, Zhang DD. Anti-ferroptotic effects of Nrf2: Beyond the antioxidant response. Mol Cell. 2023;46(3):165–75. 10.14348/molcells.2023.0005 PMC10070163 36994475

