# Supplementary material for: Ferroptosis involves in Schwann cell death in diabetic peripheral neuropathy
Source: Open Med (Wars). 2023 Oct 9;18(1):20230809. doi: 10.1515/med-2023-0809 (PMC10566555; doi:10.1515/med-2023-0809)
Supplement: Supplementary Figure [file med-2023-0809-sm.pdf]

Supplementary material

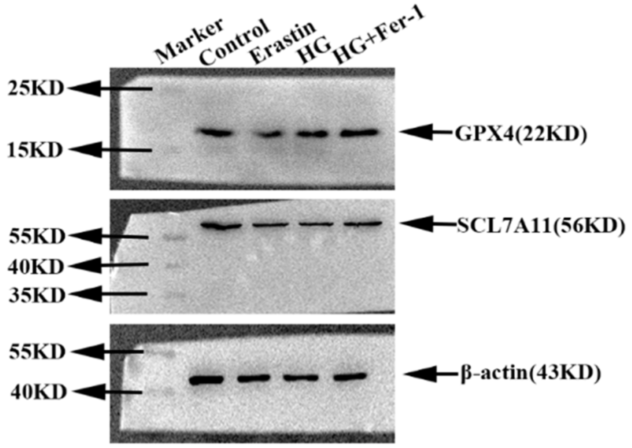

Figure S1: Original image of WB in Figure 2b.

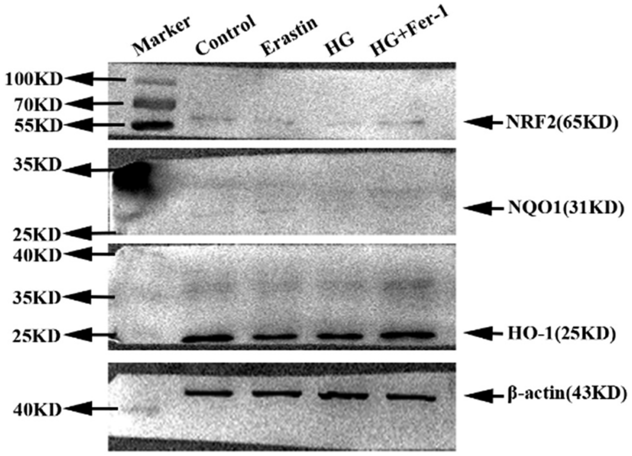

Figure S2: Original image of WB in Figure 3a.

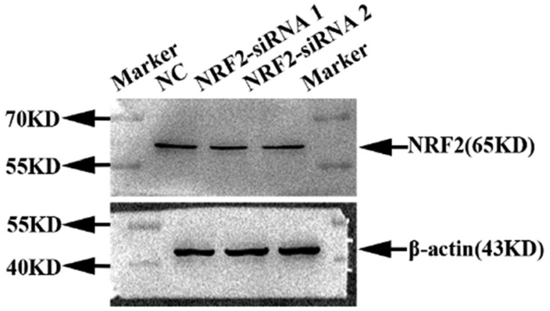

Figure S3: Original image of WB in Figure 3c.

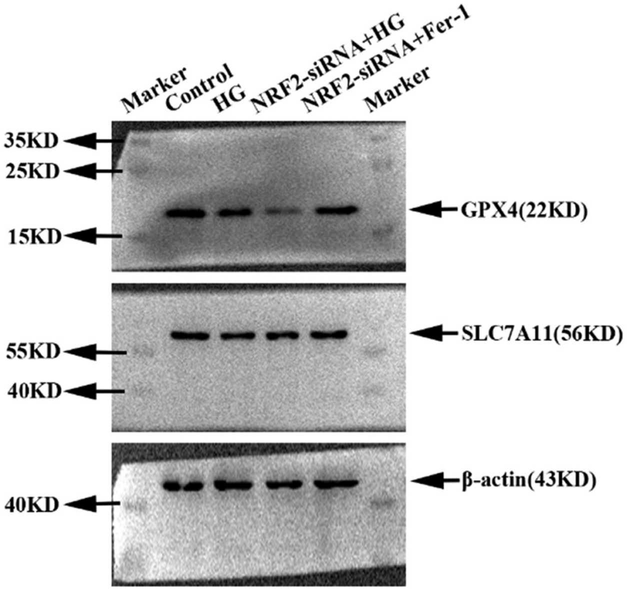

Figure S4: Original image of WB in Figure 3g.
